# Strict retroelement regulation is frequently lost following cancer transformation and generates a promising reservoir of cancer biomarkers

**DOI:** 10.1186/s13100-025-00376-7

**Published:** 2025-10-03

**Authors:** Eric Russ, Sergey Iordanskiy

**Affiliations:** 1 Department of Pharmacology & Molecular Therapeutics, Uniformed Services University of the Health Sciences, Bethesda, MD 20814 USA; 2 The Henry M. Jackson Foundation for the Advancement of Military Medicine, Bethesda, MD 20817 USA; 3 The American Genome Center (TAGC), Collaborative Health Initiative Research Program, Uniformed Services University of the Health Sciences, Bethesda, MD 20814 USA; 4 Graduate Program of Cellular and Molecular Biology, Uniformed Services University of the Health Sciences, Bethesda, MD 20814 USA; 5 Armed Forces Radiobiology Research Institute, Uniformed Services University of the Health Sciences, Bethesda, MD 20814 USA; 6 Department of Pediatrics, Stanford University, Palo Alto, CA 94304 USA

**Keywords:** Retroelements, HERV, HML-2, LINE1, Tumorigenesis, Embryonic development, HERV-H, Cancer biomarkers

## Abstract

**Background:**

Retroelements are repetitive sequences that comprise 42% of the human genome and are strictly regulated through various epigenetic mechanisms. Examining retroelement expression on a locus-specific level in relation to cancer can uncover distinct disease signatures.

**Results:**

Using over 5000 RNA-sequencing samples, we assessed retroelement transcription across 23 tissue systems, 159 cell types, 1019 cancer cell lines, and cells isolated from various stages of embryogenesis using the specialized software tool, Telescope. In healthy individuals, 11,388 retroelements were found to be actively transcribed and dynamically regulated in a tissue- and cell type-dependent manner. Using the adult human body as a reference, we observed that 94% of cancer cell lines displayed elevated transcription of at least one cancer-specific retroelement, providing a three-fold larger reservoir of cancer biomarkers (1182) than our comparable analysis of annotated protein-coding genes (338). The precise retroelements that were transcribed following tumorigenesis were influenced by the originating location, with cancers of the blood, lungs, and soft tissue displaying the highest origin-specific activation. Moreover, nearly half of the cancer-specific retroelement loci, mostly from the HERV-H family, were found to be expressed during early embryonic development.

**Conclusions:**

Our data demonstrate that elevated transcription of certain tissue-specific and embryonic retroelements can be considered as a hallmark of tumorigenesis.

**Supplementary Information:**

The online version contains supplementary material available at 10.1186/s13100-025-00376-7.

## Background

Retroelements represent a substantial portion of the human genome (42.2%) and are divided into two broad groups based on the presence of a long-terminal repeat (LTR) region [1]. The non-LTR retroelements (33.9%) consist of short interspersed nuclear elements (SINEs), long interspersed nuclear elements (LINEs), and processed pseudogenes. The LTR retroelements (8.3%) are derived from ancient retroviruses, termed endogenous retroviruses (ERVs), that were transmitted in a vertical fashion following the infection of an ancestral germ cell. Through millions of years of evolution, human ERVs (HERVs) underwent negative selection and accrued inactivating mutations [2]. However, a portion of HERVs still retain transcriptional activity and in some cases, protein-coding capacity [3–7]. Potentially harmful HERVs are strictly regulated in non-diseased tissues through various epigenetic mechanisms, but there is an extensive association between the accumulation of HERV-derived products and a diverse list of pathologies, especially those related to inflammation or cancer [8–10]. Despite this clear negative relationship between HERV activation and health, our understanding of individual proviruses and their activation in pathological conditions remains limited.

Due to the sequence homology of closely related retroelements, traditional techniques such as RT-qPCR and immunochemistry methods cannot efficiently distinguish between individual loci on the same scale as they can with annotated protein-coding genes. Despite exceptions for some distinct loci, investigations of retroelement expression have generally been restricted to the family or subfamily level. Initially, there were several meaningful attempts to overcome this limitation and study retroelements on a locus-specific level, with mixed results [11–14]. Demonstrated by Flockerzi and coauthors in their call to arms for a HERV transcriptome project, it is possible to examine the HERV-K (HML-2) subfamily using a laborious and imperfect process of cloning and sequencing individual HML-2 transcripts [11]. This approach and subsequent revisions were instrumental in demonstrating the dynamic nature of retroelement expression, but never gained widespread popularity, likely due to the limited information obtained compared to the resources required [12–14].

In light of the read length limits of early RNA-sequencing (RNA-seq) technology (25-36 bp), multi-mapped reads (reads that map to more than one genomic coordinate) were discarded due to the added complexity compared to uniquely mapped reads (reads that map to a single genomic coordinate) [15]. As a result, retroelements were not included in initial RNA-seq studies and were sparsely considered until the emergence of specialized software tools which take advantage of modern improvements to read length, sequence accuracy, and read depths to enable retroelement quantification on a locus-specific level [16, 17]. At present, one of the most commonly used software tools to analyze retroelement expression is Telescope, which implements a Bayesian statistical model to reassign ambiguously mapped reads with high efficiency based on the proportion of uniquely mapped reads [17]. Indeed, a recent analysis of psychiatric patients revealed that the implementation of Telescope into transcriptome-wide studies can reveal novel risk factors [18].

As retroelements transition into a standard component of the RNA-seq pipeline, a dedicated retroelement database is required for researchers to access, probe, and compare data throughout a broad spectrum of contexts. Herein, we present a comprehensive exploration of the retro-transcriptome, starting with an analysis of the RNA Atlas, a collection of paired PolyA RNA-seq and Total RNA-seq samples for non-diseased cell types and tissues across the human body. Previous studies relied on the Genotype-Tissue Expression (GTEx) RNA-seq dataset, which was designed to detect protein-coding genes and therefore only includes polyadenylated transcripts [19, 20]. In stark contrast to protein-coding genes, our results suggest that approximately 75% of the retro-transcriptome is non-polyadenylated and 90% is retained within the nucleus. In our study, we did not analyze the differences between independent expression (the retroelement is transcribed as its own distinct transcript) and dependent expression (the retroelement is embedded within the parental transcript) when quantifying expression levels. Nevertheless, many retroelements were exclusively expressed in a particular cell-type or tissue system, suggesting that transcription of some retroelements depends on their own or host gene regulatory sequences or they are subject to epigenetic blocking that results in context-specific expression. To identify cancer-specific retroelements and to build a catalog that supports researchers in selecting the optimal cell line(s) for phenotypic investigations, we analyzed the retro-transcriptome of 1019 cancer cell Lines available in the Cancer Cell Line Encyclopedia. We found that the retro-transcriptome was a larger source of cancer-specific markers than the transcriptome of annotated protein-coding genes. Surprisingly, 94.7% of cancer cell lines express at least one cancer-specific retroelement, with most tissue systems uniquely expressing at least five cancer-specific retroelements. Lastly, we assessed retroelement expression during embryonic development and identified potential biomarkers of stemness that are exclusive to cancer compared to the adult human body. These findings demonstrate that reactivated retroelement transcription is an intrinsic characteristic of tumorigenesis and offers a rich source of innovative biomarkers and potential anti-cancer targets.

## Methods

### Publicly available datasets

All of the RNA-sequencing samples are accessible on the GEO website with the access numbers: GSE138724 (RNA Atlas), PRJNA523380 (CCLE), GSE36552 (single-cell embryogenesis), GSE63570 (ESC priming). The raw and normalized values from each dataset can be found in our database, Retroelementdb.com [21].

### RNA-sequencing data acquisition and preparation for pipelines

The data acquisition and analyses were conducted using the Ubuntu 23.10 operating system. Publicly available raw FASTQ files were downloaded from the NCBI Gene Expression Omnibus repository using the ‘prefetch’ command from the NCBI SRA Toolkit (version 3.0.10) as*.sra* files and converted into FASTQ files with the ‘fasterq-dump’ command. FASTQ files were adaptor-clipped and quality-trimmed with Trimmomatic (version 0.39). For paired-end sequencing data sets, only paired reads were kept for downstream analysis.

### GDC pipeline

The GDC mRNA pipeline analyses gene level expression with STAR (version 2.7.10b in our case) as both the aligner and the quantifier. The current pipeline is referred to as Data Release 32 (Dr32) and can be found on their website or our database. The current reference genome used by the GDC mRNA pipeline is GRCh38.d1.vd1.fa and the current GTF file is gencode.v36.annotation.gtf. The output of the GDC pipeline is a count table with four columns: a gene ID column, followed by three count columns. The three count columns represent the counts for unstranded, forward-stranded, and reverse-stranded RNA-sequencing data. Depending on the type of sequencing, the appropriate count column was extracted from each sample and combined into a single count table, containing all of the samples for the entire dataset. Since the GDC pipeline quantifies noncoding RNAs (albeit, in a relatively inefficient manner since it only keeps uniquely mapped reads), which are not assessed in this study, we specifically extracted annotated protein-coding genes from this count table. To do so, we generated an index of protein-coding genes, as identified by the “gene_types” annotation within the gencode.v36.annotation.gtf file. This was conducted in R, with the code presented in our database.

### Telescope pipeline

The Telescope pipeline analyses retroelement expression on a locus-specific level from BAM files generated by the aligner, bowtie2 (version 2.5.0–3; other alignment packages may suffice, but we did not deviate from the authors’ published pipeline, which uses bowtie2). The reference genome hg38 from the UCSC Genome Browser website was used to build the reference index with the bowtie2-build command. To generate BAM files, we followed the settings suggested by the authors of Telescope. Then we used Telescope to quantify retroelement expression. Similarly, to the GDC pipeline, the count column was extracted from each sample and combined into a single count table, containing all of the samples for the entire dataset.

### Analysis of gene or retroelement expression

This study analyses two sources of RNA: annotated protein-coding genes and retroelements (specifically, HERVs and L1s). To normalize both sources of RNA together, the table generated from the GDC pipeline was combined with the table from the Telescope pipeline (GDC pipeline on top, Telescope pipeline on bottom) to produce a single count table. Read counts were then imported into R and analyzed with edgeR to normalize the raw data into TPM values and to determine the fold-changes and p-values (when applicable). A limitation of this process is that exonic reads from the GDC pipeline, which overlap with retroelements detected by the Telescope pipeline, are double-counted and may influence the detection of genes or retroelements that fall near our defined TPM cutoff. Detectable expression was defined as > 1 TPM in at least one sample. This value implies that a transcript is present at least once out of one million transcripts present in the cell (single-cell RNA-seq) or cell population (bulk RNA-seq). While we chose a TPM cutoff of > 1, the value may be increased or decreased, depending on the purpose of the analysis and the quality of the expression data. To perform your own analysis, we offer the raw count tables and the non-filtered TPM count tables in our database.

### Determination of PolyA:Total TPM ratio

To compare the relative rate of polyadenylation between retroelements and annotated protein-coding genes, we calculated the PolyA:Total TPM ratio by dividing the TPM value obtained from PolyA RNA-seq by the corresponding TPM value from Total RNA-seq for the same sample, as shown below:

$$\text{PolyA}/\text{Total ratio}=\frac{{\text{TPM}}_{\text{PolyA RNA}-\text{seq}}}{{\text{TPM}}_{\text{Total RNA}-\text{seq}}}$$

For analyses across the human body, only retroelements detectable by PolyA RNA-seq (TPM > 1) in at least one sample were included. In these cases, the average TPM value across the entire sequencing platform (e.g., PolyA RNA-seq) was used to calculate the ratio. For analyses of a specific cell type, only the paired PolyA RNA-seq and Total RNA-seq TPM values for that individual cell type were included.

## Results

### Building a retro-transcriptome database across the human body, cancer cell lines, and embryonic development

**Fig. 1:**
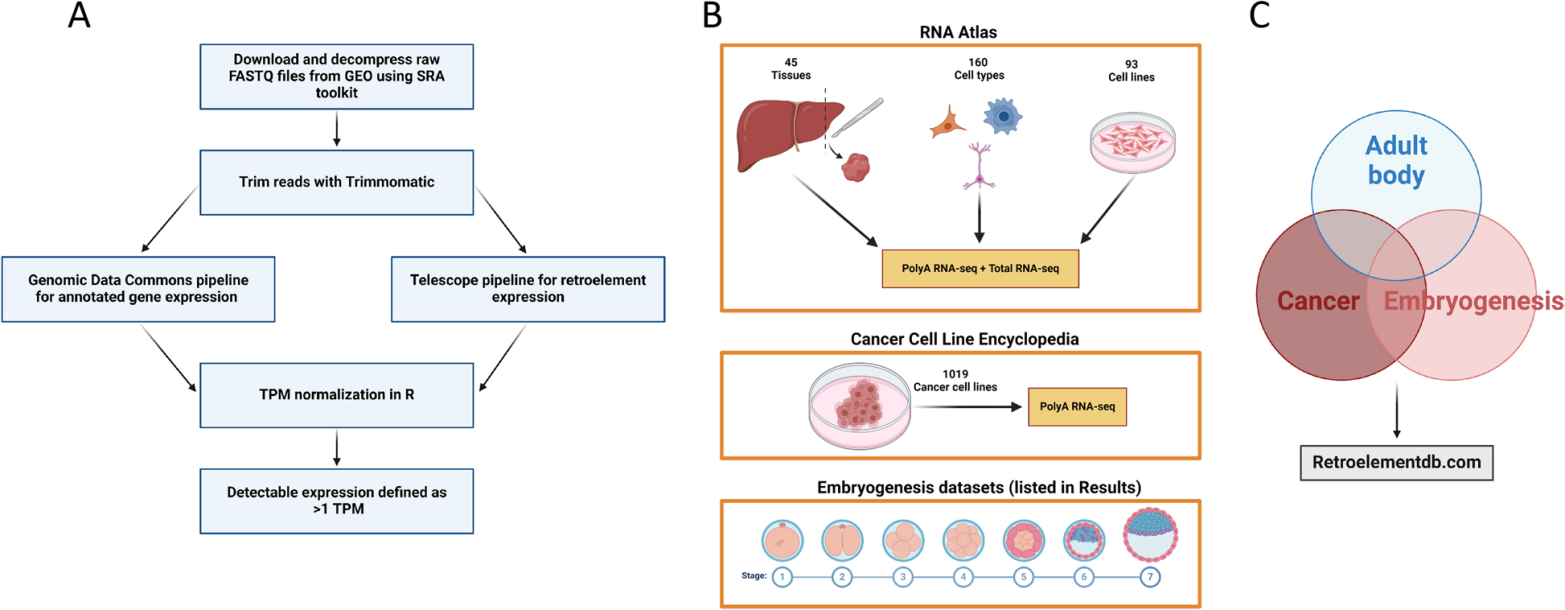
Building a retro-transcriptome database across the human body, cancer cell lines, and embryonic development. **A** Schematic overview of the Genomic Data Common (GDC) pipeline (mRNA quantification) and Telescope pipeline (retroelement quantification) for RNA sequencing data analysis. **B** Key datasets used to assess retroelement expression in different contexts. **C** The three main contexts (non-diseased samples, cancer cell lines, and embryogenesis) that are compared in this study. All findings from each analysis and figure are available in our database, Retroelementdb.com [21]. GDC, Genomic Data Commons; GEO, Gene Expression Omnibus; SRA, Sequence Read Archive; TPM, transcripts per million

As outlined in Fig. 1A, we followed two standard RNA-sequencing pipelines. The first pipeline is from the Genomic Data Commons (GDC) and is designed for mRNA quantification [22]. The second pipeline is called Telescope, available on their GitHub page, and is a specialized software tool designed for quantifying retroelement expression (including HERVs and LINEs) on a locus-specific level [17].

The datasets used in this study to assess the retro-transcriptome across the human body, cancer, and embryogenesis are publicly available on the Gene Expression Omnibus (GEO) website (Fig. 1B, C). One of the largest RNA-seq datasets for non-diseased tissues is the RNA Atlas (GSE138734) [23]. This dataset includes sequencing data for 45 tissues, 159 cell types, and 93 cell lines. A unique aspect of the RNA Atlas is that both PolyA RNA-seq and Total RNA-seq were implemented for each specimen, allowing for the detection of polyadenylated and non-polyadenylated retroelement transcripts, respectively. To identify cancer-specific retroelements and to build a catalog for functional analysis studies, we analyzed the Cancer Cell Line Encyclopedia (CCLE) from the Broad Institute. The CCLE contains genetic, transcriptomic, proteomic, and metabolomic data for over 1,000 cell lines. The transcriptomic data can be found with the accession number PRJNA523380. To identify retroelements associated with embryogenesis, we analyzed a publicly available dataset with the accession number GSE36552. This dataset was generated by performing single-cell RNA-seq on cells extracted throughout the embryogenesis process, starting with the initial oocyte to the resulting embryonic stem cells (ESCs). To understand the impact of ESC priming on the retro-transcriptome, we analyzed GSE63570.

The data generated from this study can be accessed from our newly generated database, *Retroelementdb.com* [21]. This includes the catalog of 1019 cancer cell lines and their associated retro-transcriptome, in addition to all of the raw and normalized RNA-seq count files.

### Total RNA-seq is essential for capturing the retro-transcriptomic landscape

**Fig. 2:**
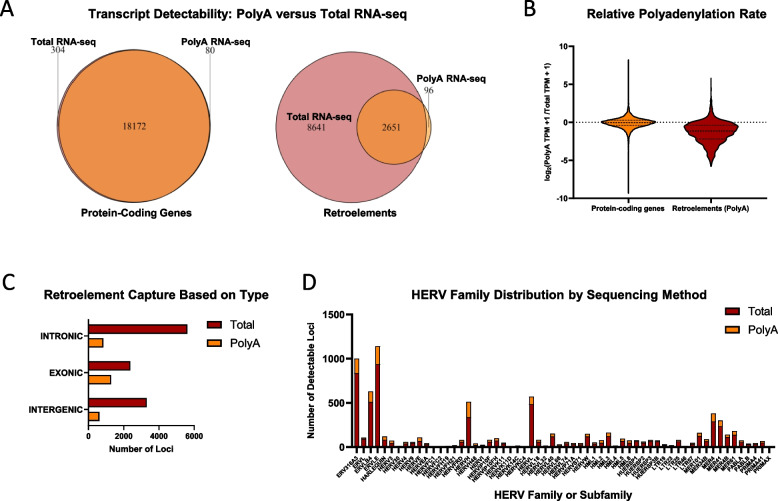
Total RNA-seq is essential for capturing the retro-transcriptomic landscape. **A** Detectability of annotated protein-coding genes and retroelements with either Total RNA-seq or PolyA RNA-seq. A total of 45 tissues and 159 cell types, available in the RNA Atlas database were included in this analysis. Detectability is defined as a TPM > 1 in at least one sample. **B** Relative polyadenylation rate of annotated protein-coding genes and retroelements. For this analysis, only retroelements that are detectable with PolyA RNA-seq were included. **C** Number of intronic, exonic or intergenic retroelements that are captured with either sequencing method. **D** HERV family and subfamily distribution, depending on the sequencing method

We first sought to build a reference list of retroelements expressed in non-diseased conditions. For the purposes of this study, our analysis does not distinguish between independent and dependent (the retroelement is a part of a parent transcript) retroelement expression, as the retroelement sequence is transcribed and present within the cell in either case. Across the human body, transcription of 18,556 annotated protein-coding genes and 11,388 retroelements were detected in at least one cell type or tissue (Fig. 2A, Supplemental Table S1). To prioritize the sequencing of certain types of RNAs, one of two types of RNA clean-up are typically performed prior to RNA-sequencing: 1) polyA-enrichment, to selectively isolate polyadenylated transcripts or 2) rRNA-depletion, to actively remove rRNA and retain all other types of RNA. Depending on the type of RNA clean-up that is used, the resulting sequencing process is referred to as either PolyA RNA-seq or Total RNA-seq, respectively. Previous analyses of the retro-transcriptomic landscape relied on the GTEx dataset, which was designed for protein-coding genes and therefore only implements PolyA RNA-seq [19, 20, 24]. While nearly every protein-coding gene could be detected with either PolyA RNA-seq or Total RNA-seq, polyA-enrichment removed approximately 75% of the retro-transcriptome (defined by the detection of individual loci), according to our analysis.

Among the retroelement loci that are detected via PolyA RNA-sequencing, an important question is whether they experience similar or dissimilar rates of polyadenylation compared to annotated protein-coding genes. To estimate if a retroelement is efficiently polyadenylated or consistently preserved within a polyadenylated transcript (presumably, mRNA(s) derived from their host gene), we calculated the ratio of the transcript per million (TPM) values obtained from PolyA RNA-seq and Total RNA-seq (detailed further in Methods). To provide context, annotated protein-coding genes generally have a PolyA:Total TPM ratio of one in our analysis (or a log_2_ ratio of zero), consistent with a previous analysis of the RNA Atlas (Fig. 2B, Supplemental Table S2) [23]. Based on this methodology, the rate of polyadenylation for retroelements or retroelement-containing RNAs appears to be markedly lower than that of annotated protein-coding genes. Specifically, the median PolyA:Total TPM ratio is 0.45 for retroelements compared to 0.97 for protein-coding genes. Depending on the retroelement, this could either reflect a weak or deleted polyadenylation signal or, for retroelements embedded within host transcripts, indicate alternative splicing events that generate a mixed population of mRNAs – some retaining and others lacking the embedded retroelement [25].

Retroelements can exist as independent genomic elements, known as intergenic loci, or they can reside within annotated genes. Based on the retroelement’s genomic coordinates in relation to annotated genes, Telescope assigns each retroelement as either intergenic, intronic or exonic. All types of retroelements were inefficiently captured by PolyA RNA-seq, but with a bias in favor of exon-residing retroelements compared to intronic or intergenic loci (Fig. 2C, Supplemental Table S3). Specifically, in reference to the loci detected by Total RNA-seq, PolyA RNA-seq could only detect 19% of the transcribed intergenic loci, 54% of the exonic loci, and 15% of the intronic loci. Of the retroelement loci absent from PolyA-enriched Libraries, approximately 31% were intergenic, 56% intronic, and 12% exonic. These patterns suggest that many retroelements are either expressed as independent, non-polyadenylated transcripts or arise from the transcription of their host gene and are subsequently spliced out during RNA maturation. Thus, Total RNA-seq is required to efficiently capture the retro-transcriptomic landscape, similarly to its importance in studies focused on long noncoding RNAs (lncRNAs) [26].

Among the sixty HERV families, with each HERV-K subfamily counted as an independent family for simplicity, the highest number of expressed HERV loci were from the ERVLE (937 loci), ERV316A3 (836 loci), ERVLB4 (510 loci), HERVL (485 loci), and HERVH (338 loci) families (Fig. 2D, Supplemental Table S3). None of the families displayed an enrichment for polyadenylation, with most families fluctuating between 10–30% of their detectable loci from Total RNA-seq also being detectable via PolyA RNA-seq. Interestingly, at least one member from each family was detectably expressed, an observation that relies on the use of Total RNA-seq and further highlights its utility for retroelement-focused studies.

### Retroelement expression is tissue- and cell lineage-dependent

Retroelement expression is known to be tightly regulated, with the expression patterns of individual loci shaped by both the tissue system (e.g., kidney versus heart) and alterations to the physiological context (e.g., resting versus activated states) [20, 27, 28]. However, analyses that incorporate individual cell types across the human body, rather than relying primarily on bulk tissue samples, remain limited. To address this, we surveyed retroelement expression across multiple organs (e.g., lung, kidney, placenta), broader systems (e.g., CNS, endocrine), and cell types, as defined by the RNA Atlas; paired PolyA and Total RNA-seq data were available for nearly every sample and included in our analysis (Supplemental Table S4).

**Fig. 3:**
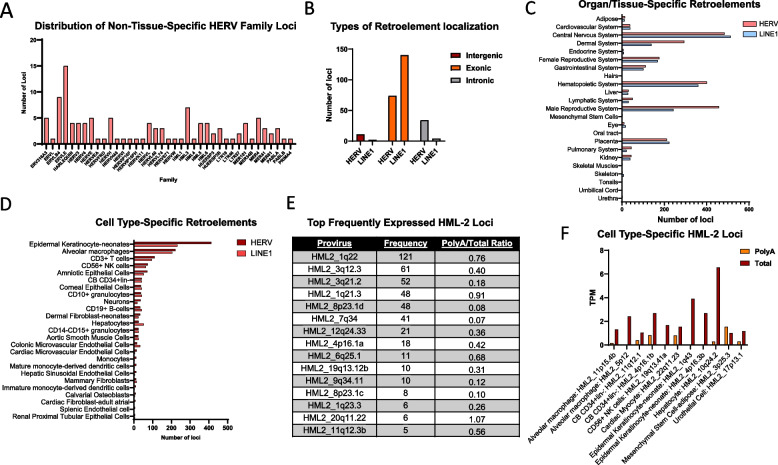
Cell type-specific and non-specific retroelements. **A** Family distribution of retroelements that are expressed in at least one sample from each tissue system. **B** Types (intergenic, exonic or intronic) of retroelements that are expressed in different tissues. **C** Retroelements that are exclusively expressed in one organ/tissue system. The organ/tissue systems were previously identified by the RNA Atlas. **D** Retroelements that are exclusively expressed in one cell type (159 cell types in total as indicated in RNA Atlas). **E** The top 15 frequently expressed HERVK HML-2 loci among 159 cell types. **F** Polyadenylated and total RNA derived from HML-2 loci with cell type-specific expression. All data in panels A-F are obtained from the RNA Atlas dataset analysis

Using a TPM cutoff of > 1, we identified 265 retroelements that were detected in at least one cell type or bulk tissue sample from each of the 23 defined tissue systems (Fig. 3A). Relevant information pertaining to these recurrently expressed loci (family, genomic relation to canonical genes, predicted coding capability, etc.) is provided in Supplemental Table S5. Most of the recurrently expressed retroelements (detectable in 23/23 tissue systems) are either intron- or exon-residing, with only 13 intergenic loci (Fig. 3B). The cross-tissue prevalence of the 252 intronic and exonic retroelements can be partially explained by their presence within housekeeping genes or other genes that are commonly expressed across tissues. Specifically, we observed that 220 of these retroelements reside in host genes with similarly broad expression patterns (> 1 TPM in 23/23 tissue systems; Supplemental Table S6). While some of these host genes do not have a clearly defined biological role, many encode core cellular machinery that is expected to be ubiquitous. These include ribosomal proteins and RNA-processing factors essential for protein synthesis (e.g., RPS23, MRPS5, RBM26), enzymes required for mitochondrial metabolism and energy production (e.g., SDHA, HADHB, NNT), and regulators of protein quality control and turnover (e.g., UBR5, HUWE1, USP15). Others participate in protein trafficking and folding, such as ER-resident chaperones and vesicle transport proteins (e.g., SEC63, LMAN1, DNAJC10). These contexts Likely explain the recurrent detection for the majority of our observed 265 universally expressed retroelements; however, they do not account for the recurrent expression of intergenic retroelements or of intronic/exonic retroelements whose host genes are not broadly expressed. Future studies are needed to elucidate whether these retroelements are biologically relevant.

By comparing the retro-transcriptome of each tissue system, we identified 4343 retroelement loci with apparent tissue-specific expression (Fig. 3C, Supplemental Table S5). The top five most enriched families among the tissue-specific HERVs are ERV316A3 (396 loci), ERVLE (392 loci), ERVLB4 (201 loci), HERVH (201 loci), and HERVL (191 loci) (Supplemental Table S7). Interestingly, the abundance of tissue-specific retroelements correlates with the abundance of tissue-specific canonical genes (Supplemental Fig. 1A, B). Moreover, there is an equal distribution of tissue-specific HERVs and L1s, except for the 2:1 ratio observed in the dermal and male reproductive systems. This could indicate selective activation of HERVs in these tissues, but additional studies are needed to understand these findings.

In the 159 cell types examined, we identified 2334 retroelements with cell type-specific expression (Fig. 3D, Supplemental Table S5). Surprisingly, neonatal epidermal keratinocytes exhibited the most unique retro-transcriptome. It should be noted that fetal and adult epidermal keratinocytes were also included in this analysis and that they only displayed six uniquely expressed retroelements combined. This suggests that within the same cell lineage, at different key points in the developmental process, retroelements may be either selectively induced or repressed. As suggested by Fig. 3C and D, the adult hematopoietic system is a source for many cell types with a high abundance of uniquely expressed retroelements, including alveolar macrophages, T cells, natural killer (NK) cells, B cells, and others. This could be due to the relatively specialized roles that each of these cell types are designed to perform, leading to a large quantity of active transcription factors, triggered by various signaling pathways, and distinct epigenetic environments within a single tissue system.

Given that the HML-2 subfamily is suggested to have a high degree of biological activity [6, 8, 29, 30], we examined them more closely (Fig. 3E). Interestingly, the HML-2 provirus located at 1q22 (HML2_1q22) is expressed in 121 out of 159 cell types. We previously demonstrated that HML2_1q22, otherwise known as HERV-K102, is selectively induced following interferon gamma and may participate as a downstream signaling component to enhance pro-inflammatory signaling [28]. Other HML-2 proviruses are frequently expressed, including HML2_3q12.3, HML2_3q21.2, HML2_1q21.3, HML2_8p23.1d, and HML2_7q34. While a previous analysis of the HML-2 subfamily and its LTRs for transcription factor motif enrichment does not clearly explain the recurrent expression patterns observed for these loci [20], HML2_7q34 can be explained due to it residing within the housekeeping gene, SSBP1. However, none of the top five most frequently expressed HML-2 proviruses are associated with a known protein-coding gene. Additionally, we observed that most of the frequently expressed HML-2 proviruses had a PolyA:Total TPM ratio below 0.50, suggesting either a low rate of polyadenylation (if transcribed independently) or a high rate of removal during RNA maturation (if transcribed through their host gene).

A total of 11 HML-2 proviruses were cell type-specific, including HML2_11p15.4b, HML2_5p12, HML2_11q12.1, HML2_4p16.1b, HML2_19q13.41a, HML2_22q11.23, HML2_1q43, HML2_4p16.3b, HML2_10q24.2, HML2_3p25.3, HML2_17p13.1 (Fig. 3F). However, most of them only reached the detection threshold (TPM > 1) using Total RNA-seq. It is possible that some of these proviruses are only expressed in distinct cells of certain individuals or that they are expressed in other cell types, depending on the individual. For instance, HML2_11p15.4b was only detected in one out of two alveolar macrophage donors. This person-to-person variability should be investigated in a larger donor population to optimize the selection of retroelements for biomarker development.

### Retroelements are mostly non-polyadenylated and are retained within the nucleus

**Fig. 4:**
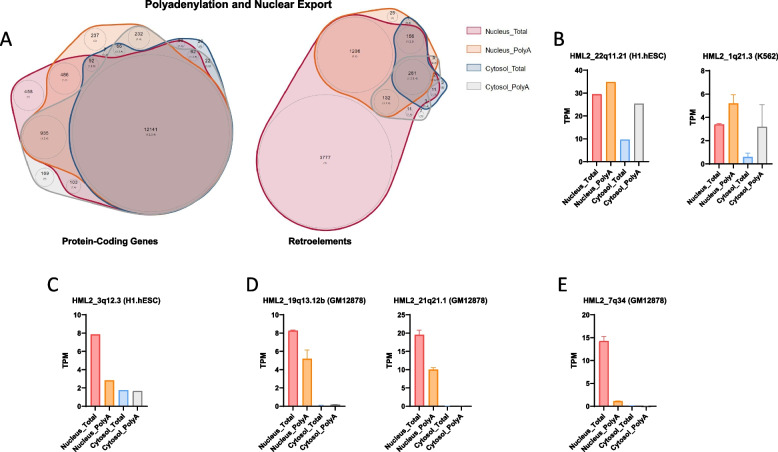
Retroelements are mostly retained within the nucleus. **A** Detectability of annotated protein-coding genes and retroelements in the nucleus and cytosol, using PolyA and Total RNA-seq. **B** An example for retroelements with a similar polyadenylation and nuclear export profile as annotated protein-coding genes. **C** An example for retroelements with inefficient polyadenylation and inefficient nuclear export. **D** An example for retroelements that are inefficiently polyadenylated and retained within the nucleus. **F** An example for retroelements that are non-polyadenylated and retained within the nucleus. All data in panels A-F are obtained from the RNA Atlas dataset analysis; the ENCODE dataset contained one or two samples per condition

RNA localization can influence its function and targetability [31–34]. Since most retroelement transcripts are non-polyadenylated and may therefore exhibit a reduced capacity to translocate from the nucleus to the cytoplasm, we examined retroelement RNA localization using a dataset (GSE30567) from the Encyclopedia of DNA Elements (ENCODE) [35]. This dataset was generated by performing PolyA RNA-seq and Total RNA-seq on the nuclear and cytosolic fractions from six cell lines: the lymphoblastoid lines GM12878 and K562, the cervical adenocarcinoma line HeLa-S3 (a subclone of the HeLa line), the hepatoblastoma line HepG2, the embryonic stem cell line H1-hESC, and primary umbilical vein endothelial cells (HUVEC). Consistent with the RNA Atlas dataset, only 25% of retroelements were polyadenylated (Fig. 4A, Supplemental Table S8). A smaller fraction, slightly over 10%, were identified in the cytosol. It should be noted that these percentages may not be entirely precise, since an unknown number of retroelements/transcripts are spliced out and rapidly degraded during RNA maturation, and our analysis does not distinguish between independent and dependent transcription. Interestingly, polyadenylation was not a requirement for the nuclear export of some retroelement RNAs.

Despite most retroelements being non-polyadenylated and retained within the nucleus, four main patterns of polyadenylation and nuclear export can be observed, with members of the HML-2 subfamily serving as examples (Fig. 4B-E). Although comparing TPM values across sequencing methods and cellular fractions is not entirely precise, the TPM patterns for the proviruses HML2_22q11.21 and HML2_1q21.3 resemble that of beta actin (Fig. 4B, C; Supplemental Fig. S2). It is noteworthy that while HML2_22q11.21 is technically classified as exonic for several annotated genes, the “host” genes were undetected in our analysis. This could suggest that HML2_22q11.21 retains some level of biological activity and drives expression through its own long-terminal repeat (LTR) promoter. Previous evidence supports this, showing that HML2_22q11.21 encodes a functional Gag protein and that its transcripts are selectively packaged into viral-like particles released from the human germ cell tumor line, Tera-1 [36, 37]. Additionally, HML2_22q11.21 was shown to recruit the transcription factor sex-determining region Y-box 2 (Sox2) and thereby act as an enhancer for a nearby gene, proline dehydrogenase (PRODH) [38]. Sox2 is critical for embryonic development and the maintenance of stem cell pluripotency, which may explain why this provirus was detected in the embryonic cell line H1-hESC but not in the other five lines nor within the RNA Atlas of non-diseased samples [39]. The HML2_1q21.3 provirus is intergenic and widely expressed throughout the human body, but its potential function is unknown. These results suggest that a portion of retroelements drive their own expression, are polyadenylated, and undergo efficient nuclear export. Future studies are required to elucidate their potential functions.

Retroelements such as HML2_3q12.3 are inefficiently polyadenylated but still undergo nuclear export (Fig. 4C). Other retroelements, including HML2_19q13.12b and HML2_21q21.1, are polyadenylated to an extent but retained within the nucleus (Fig. 4D). Lastly, HML2_7q34 represents a standard intron-residing provirus, which is expressed as part of an annotated protein-coding gene (in this case, SSBP1) but spliced-out and rapidly degraded during RNA maturation (Fig. 4E). In general, the four patterns of retroelement polyadenylation and nuclear export can be defined as: 1) efficient polyadenylation and nuclear export, 2) moderate or inefficient polyadenylation but still capable of nuclear export, 3) moderate or inefficient polyadenylation and retained within the nucleus, and 4) non-polyadenylated and retained within the nucleus. Taken together, these data indicate that retroelements are largely retained within the nucleus and that while polyadenylation may support nuclear-cytoplasmic translocation, it does not necessarily entail that nuclear export occurs for retroelement RNAs. How this influences the detection of disease-associated retroelements in the blood or other bodily fluids remains unclear. However, it can be assumed that the spectrum of these retroelements will be shifted towards those whose RNA is detected in the cytoplasm.

### Retroelements constitute the major ratio of cancer-specific transcripts

To identify retroelements that are specifically expressed in cancer cells, we analyzed PolyA RNA-seq data for all 1019 cancer cell lines included in the CCLE dataset. Despite the limitation that PolyA RNA-seq cannot capture the majority of transcribed retroelements, the CCLE contains the largest collection of cancer cell lines by a considerable degree. As a reference for a non-diseased adult human body, we used the RNA Atlas dataset illustrated in Fig. 1 and excluded samples of embryonic or placental origin. All genes and retroelements identified as expressed within non-diseased samples, regardless of whether they were detected via PolyA or Total RNA-seq, were included to ensure cancer-specificity when cross-referenced with the CCLE dataset.

**Fig. 5:**
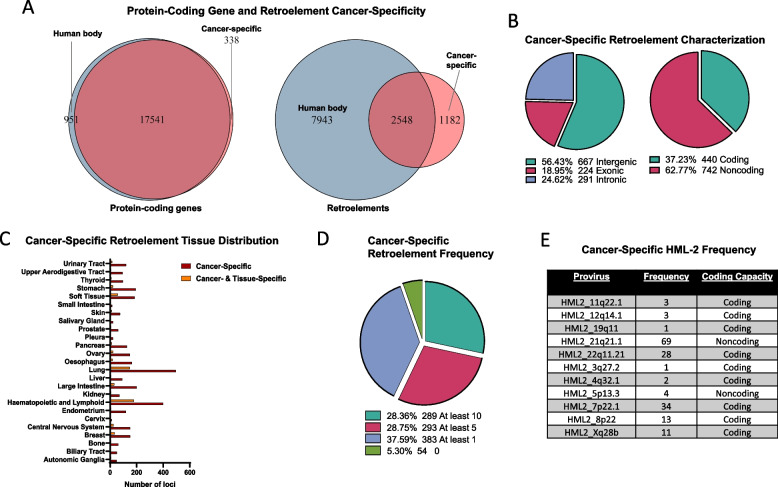
Retroelements constitute the major ratio of cancer-specific transcripts. **A** Identification of cancer-specific retroelements. Non-diseased samples from the RNA Atlas, excluding those of embryonic or placental origins, were used as a reference. **B** Types (intergenic, exonic or intronic) and coding capacity of retroelements that are specific to cancer. **C** Distribution and uniqueness of cancer-specific retroelements in different tissues. Tissues are defined as the predicted origin for the cancer cell line and were identified by the Cancer Cell Line Encyclopedia. **D** Frequency of cancer-specific retroelement expression across all 1019 cancer cell lines. **E** Frequency of expression for cancer-specific HML-2 loci and their coding capacity

From this comparison, we found that 338 annotated protein-coding genes were cancer-specific, in contrast to 1182 retroelements (Fig. 5A). Among the cancer-specific retroelements, 573 were HERVs and 609 were L1s, with a notable enrichment of HERVH loci (274) across the HERV families (Supplemental Table S9). For reference, the family with the second highest number of cancer-specific loci was ERVL, with only 24 proviral loci. Interestingly, the majority of the cancer-specific retroelements were intergenic (667; 56.4%), and a large portion are suggested to retain or possess partial coding capacity (440; 37.2%) according to a previous Coding-Non-Coding Identification Tool (CNIT) analysis performed by the authors of Telescope (Fig. 5B) [40]. To provide additional context, while a similar proportion (50.8%) of the tissue-specific retroelements were intergenic, a substantially smaller fraction (4.8%) of them are predicted to have coding capacity. This strikingly high proportion of protein-coding retroelements among the cancer-specific transcripts may reflect bias introduced by PolyA selection (as the tissue-specific retroelements were identified through a combination of PolyA and Total RNA-sequencing), or it may indicate the preferential activation of retroelements with particular features (e.g., coding capacity) during or following tumorigenesis. Replicating the CCLE dataset with Total RNA-seq would likely resolve this distinction and uncover an even greater repertoire of cancer-specific retroelements.

The cell lines from the CCLE are classified as originating from one of twenty-five lineages (for simplicity, we will refer to them as tissues; refer to Supplemental Table S10 for each tissue and associated cell line). The tissues, as defined by the CCLE, are not entirely congruent with the tissues defined by the RNA Atlas and should not be viewed as inherently cross-compatible. We found that cancers from every tissue (using the CCLE’s classifications for tissues) expressed cancer-specific retroelements (Fig. 5C; Supplemental Table S11). All tissues except for two (salivary gland and cervix) expressed at least one retroelement that is both cancer- and tissue-specific, meaning that the retroelement is 1) only detected in cancer cells and 2) only detected in cancer cell lines from one tissue (Fig. 5C; Supplemental Table S11). Among the 1019 cell Lines, nearly 95% expressed at least one cancer-specific retroelement and over 28% expressed more than ten (Fig. 5D). Twelve retroelements were transcribed in at least 10% of cancer cell lines, including HERVH_6q22.1c (33.1%), L1FLnI_5q21.2a (21.6%), L1FLnI_14q12oa (19.6%), HERVH_6q12f (16.8%), HERVH_3q13.11 (15.4%), HERVH_6q24.2 (13.3%), HERVH_Xp22.32a (13.3%), HERVH_1q41f (13.0%), L1FLnI_22q11.22b (12.2%), L1FLnI_Xq25oa (11.3%), HERVH_8q21.3b (10.9%), HERVK11_8q11.1 (10.9%) (Supplemental Table S11).

Eleven HML-2 proviruses were found to be cancer-specific (Fig. 5E). Nine are predicted to be protein-coding: HML2_11q22.1, HML2_12q14.1, HML2_19q11, HML2_22q11.21, HML2_3q27.2, HML2_4q32.1, HML2_7p22.1, HML2_8p22, and HML2_Xq28b (Supplemental Table S12). While the proviruses HML2_22q21.1 and HML2_5p13.3 are predicted to be noncoding, they may have certain regulatory functions inherent to lncRNAs or can be used as cancer biomarkers. The top five most commonly expressed HML-2 loci are not associated with any known protein-coding genes. Among HML-2 proviruses associated with coding genes, HML2_7q34 is an intronic provirus that resides within SSBP1, a housekeeping gene related to mitochondria biogenesis. HML2_12q24.33 is embedded within ZNF140, a zinc finger protein with relatively ubiquitous expression across tissues (according to the Human Protein Atlas [41]).

To verify our findings, we compared these eleven HML-2 proviruses with an analysis of the GTEx dataset that focused solely on the HML-2 subfamily [20]. Out of our eleven cancer-specific HML-2 proviruses, only HML2_7p22.1 and HML2_12q14.1 were detectably expressed in the GTEx dataset. Their expression in some non-diseased samples from the GTEx dataset but not in the RNA Atlas could be due to the substantially larger number of individuals that are sequenced within the GTEx dataset. Considering that the RNA Atlas and GTEx datasets are the largest collections of RNA-seq samples for non-diseased tissues, the nine proviruses that were unexpressed in both are likely to be unique to cancer. However, additional studies are needed to confirm this.

### Embryogenesis is a significant source of stemness-associated cancer-specific retroelements

**Fig. 6:**
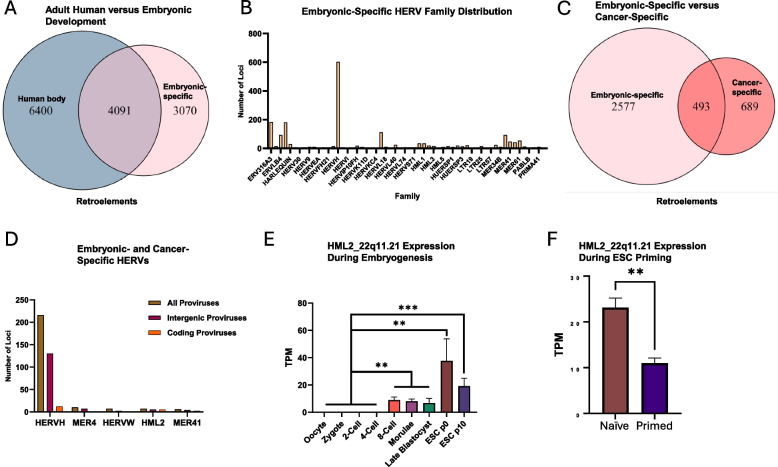
Embryogenesis is an important source of stemness-associated cancer-specific retroelements. **A** Identification of embryonic-specific retroelements. Non-diseased samples from the RNA Atlas, excluding those of embryonic or placental origins, were used as a reference. **B** HERV family distribution of embryonic-specific retroelements. **C** Comparison between embryonic-specific and cancer-specific retroelements. **D** Top five HERV families with the most dual embryonic- and cancer-specific loci. **E** Expression of the HML-2 provirus, HML2_22q11.21, during embryogenesis. **F** Expression of HML2_22q11.21 following embryonic stem cells (ESC) priming. For panels E and F, data are presented as means ± standard error of the mean (SEM). **,*P* < 0.01; ***,*P* < 0.001 according to edgeR analysis for panels E and F

ESCs are pluripotent cells that constitute the inner cell mass of the blastocyst during embryogenesis. Their self-renewal properties (the ability to proliferate indefinitely, in vitro) are reminiscent of those seen in cancer stem cells (CSCs), with many shared markers between them [42]. CSCs are a subpopulation of cancer cells with resistance to chemotherapy and radiotherapy due to various reasons, including their pluripotent nature, high expression of ABC drug transporters, efficient DNA repair machinery, and other factors [43] [44]. Consequently, CSCs are believed to be a prominent cause of cancer relapse and inhibiting the pathways that maintain CSC stemness has been shown to prevent this [44]. To identify retroelements that may have a contributing role to stemness, we used a publicly available dataset (GSE36552) to examine retroelement expression during embryogenesis on a single-cell level, with cells taken during each major step as an oocyte develops into an ESC. Consistent with earlier publications, we found that embryogenesis is a rich source of retroelements, with 7161 loci expressed in total (Fig. 6A; Supplemental Table S13) [45, 46]. Astonishingly, over 3000 retroelements are unique to embryogenesis and repressed in the adult human body, including in mesenchymal stem cells isolated from discrete adult tissues. Most of the embryonic-specific HERVs were from the HERVH family, corroborating previous evidence that members of the HERVH family are highly expressed in ESCs (Fig. 6B; Supplemental Table S14) [46, 47].

To determine if embryonic-specific retroelements are reactivated during tumorigenesis, we performed a comparison with the cancer-specific retroelements identified from the CCLE dataset (Fig. 6C). Surprisingly, 42% of the cancer-specific retro-transcriptome are reawakened retroelements that were once expressed during embryogenesis. A similar percentage can be observed for protein-coding genes, albeit at a three-fold lower quantity (493 retroelement loci; 161 protein-coding genes) (Supplemental Fig. S3). The HERVH family accounted for over 90% of the dual embryonic- and cancer-specific HERVs, with most being intergenic proviruses (Fig. 6D). For this figure, only the top five retroelement families are shown (refer to Supplemental Table S15 for a full list). Although only 12 out of the 216 HERVH proviruses are predicted to be protein-coding, previous studies have suggested that noncoding HERVH transcripts are essential for maintaining embryonic stem cell identity and pluripotency [48, 49].

Among the 35 potential protein-coding retroelements that are dual embryonic- and cancer-specific, three loci comprise greater than 94% of the sum for these transcripts during embryogenesis (HERVE_16q23.1, HML2_22q11.21, and HML4_10p15.1) (Supplemental Fig. S4A). Interestingly, HERVE_16q23.1 and HML4_10p15.1 are highly expressed during the oocyte to 4-cell or morulae cell stages, respectively, but repressed at the blastocyst stage (Supplemental Fig. S4B). Conversely, HML2_22q11.21 is repressed until the 8-cell stage and experiences a boost in mean expression as the blastocyst develops ESCs (Fig. 6E). Passaging of the fresh ESCs from p0 to p10 results in an approximately two-fold decrease in expression of HML2_22q11.21 (Fig. 6F). Unfortunately, variability of expression among the individual cells caused the differences between the blastocyst, ESCs at p0, and ESCs at p10 to be nonsignificant. To compensate for this variability, we coupled these findings with a dataset (GSE63570) that used a cell Line derived from an embryo at the 8-cell stage (ELF1) to compare the transcriptome of naïve ESCs to that of ESCs “primed” for lineage specification. As seen during the passaging of ESCs from p0 to p10, there is a decrease in HML2_22q11.21 expression during the priming of ESCs for lineage specification (Fig. 6F). Intriguingly, HML2_22q11.21 was most highly expressed in three ovarian cancer cell lines (OVMANA, IGROV-1, and OVISE), potentially making it a suitable target for future ovarian cancer research (Supplemental Table S16). Collectively, these results indicate that embryonic-specific retroelements are reawakened during the tumorigenesis process and contribute significantly to the cancer-specific retro-transcriptome. Knockout studies against the identified dual embryonic- and cancer-specific retroelements, using our catalog of retroelement expression across 1019 cancer cell lines to select the optimal line, could elucidate the specific role of these retroelements in carcinogenesis and uncover novel targets against cancer stemness.

## Discussion

Repositories of expression data are readily available for annotated genes, allowing researchers to easily assess the relationship between transcripts and pathological conditions, but a comparable resource for retroelements is notably absent [24, 41, 50]. With mounting evidence that retroelement dysregulation is a hallmark of tumorigenesis, a comprehensive database of retroelement expression in non-diseased tissues is imperative [51]. Such a database would serve as a critical reference point for assessing the specificity of retroelement transcripts to cancer and other pathological conditions. This distinction is vital for incorporating retroelements into biomarker panels or developing potential anti-cancer therapies. To address this gap, by this study we initiated the development of a retroelement expression database by analyzing the retro-transcriptome across non-diseased tissues, cancer cell lines, and cells isolated during embryonic development.

Our analysis of non-diseased tissues corroborates previous evidence that retroelements are actively transcribed and dynamically regulated in human adults [19, 20]. Context-driven expression, especially for intergenic loci, could indicate co-option events wherein the host genome recruits a retroviral gene through selective pressure to enact a beneficial biological function [52–54]. The effects of these cell type- and tissue-specific retroelements on host physiology are currently unknown, but in accordance with their noncoding disposition, we found that the vast majority, are non-polyadenylated (~ 75%) and retained within the nucleus (~ 90%). This suggests that most retroelements are either spliced-out from parent transcripts during RNA maturation or are relegated to regulatory tasks, mostly as noncoding RNAs, yet the nuclear export of a portion of retroelements implies a more intricate and diverse set of roles for the retro-transcriptome [55]. Detailed knockout studies, potentially with cell type-specific loci, are likely to elicit novel findings on the importance of retroelements in the maintenance of cell identity and function. Moreover, when designing future retroelement analyses, our findings demonstrate that total RNA-seq is required to capture the complete retro-transcriptomic landscape.

**Fig. 7:**
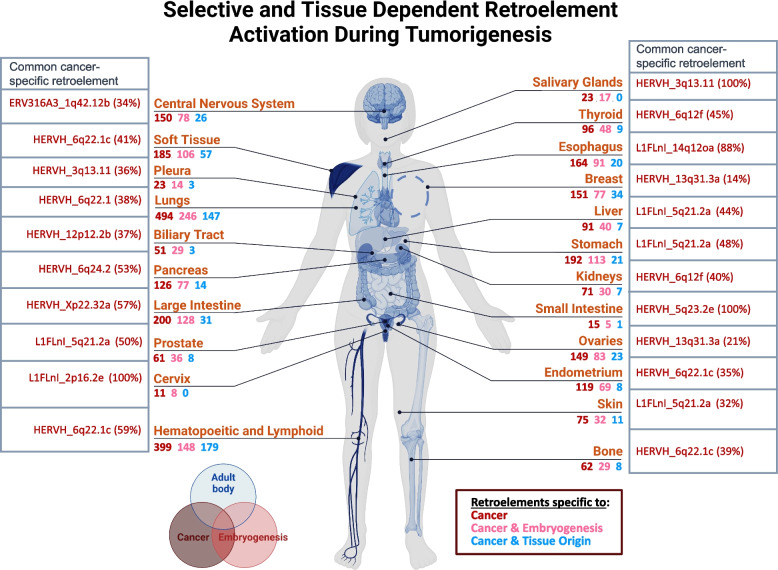
Selective retroelement activation during tumorigenesis. Tissue-related and embryogenesis-associated cancer-specific retroelements in different types of cancer: the number of retroelements for each tissue that are cancer-specific, cancer- and embryonic-specific or cancer- and tissue-specific. The most commonly expressed cancer-specific retroelement in each tissue origin is highlighted, with its frequency in the cancer cell line population represented as a percent

Our survey of 1019 cancer cell Lines from the CCLE suggests that retroelements provide a three-fold larger reservoir of cancer-specific RNA markers compared to same analysis for annotated host protein-coding genes. Considering that the CCLE dataset is based on PolyA RNA-seq and does not incorporate Total RNA-seq, our identified 1182 cancer-specific retroelements is Likely a significant underestimation that only accounts for the contribution of polyadenylated transcripts. Applying the same sequencing methodology as the RNA Atlas on a sample size as large as the CCLE would undoubtedly uncover an even larger and more diverse repertoire of cancer-specific retroelements. Even so, we found that retroelement activation during or following tumorigenesis is a nearly universal phenomenon. Over 94% of cancer cell lines were observed to express at least one cancer-specific retroelement, a substantial fraction (~ 37%) of which are predicted to be protein-coding, providing a source of potential targets for immunotherapy [56, 57]. Furthermore, most tissue systems exhibited a list of uniquely expressed cancer-specific retroelements, which would allow for biomarker panels to not only detect that a patient has cancer but also to predict its origin (Fig. 7, Supplemental Table S17). These findings hint at significant potential for retroelements as analytes for “liquid biopsies,” a noninvasive laboratory test that analyzes bodily fluids to detect and monitor cancer [58]. Although more work is needed to prove true specificity, our results support the initiation of pilot studies to investigate the level of detectability of these transcripts in the blood and other bodily fluids.

The observation that approximately 41% of the cancer-specific retroelements reflect reactivation events of embryonically expressed retroelements that undergo silencing prior to adulthood is particularly interesting. Although information on these individual loci is limited, previous evidence suggests that disruption of either HERV or LINE expression during embryogenesis negatively impacts ESC pluripotency and embryo development [48, 49, 59, 60]. These findings indicate that at least a portion of the overlapping retroelements may contribute to the stemness and plasticity characteristics of CSCs, but knockout studies are required to determine which loci have a functional relationship with malignant transformation and cancer development. Moreover, it should be noted that non-embryonic retroelements may also contribute to the manifestation or maintenance of CSCs. Specifically, the HML-2 proviruses HML2_11q22.1 and HML2_19q11 were enriched in malignant glioma cells that displayed a stem cell phenotype yet were unexpressed during embryonic development [61]. A version of Telescope that is specialized for single-cell RNA-sequencing, Stellarscope, is currently under review and will likely assist in clarifying which precise retroelements are associated with CSCs [62].

A limitation of this study is that we primarily focused on the uniqueness of a retroelement’s expression to a specific context, without assessing the relative expression or rate of polyadenylation between contexts. For instance, the intronic provirus HML2_5q33.3, located in an antisense direction within the sarcoglycan delta (SGCD) gene, was detected with Total RNA-seq in several tissues but lost during polyA enrichment (Supplemental Table S18). Based on the level of SGCD expression, it is likely that HML2_5q33.3 is spliced-out during RNA maturation and rapidly degraded in normal tissues. Conversely, this retroelement was highly expressed as a polyadenylated transcript in passage 0 of ESCs while its host gene was unexpressed (Supplemental Table S19). This suggests that HML2_5q33.3 can drive its own expression depending on the context and may mediate an embryonic-associated function, which could potentially be recapitulated in certain types of cancers. Similarly, cancer-enriched retroelements, even if they are expressed in a portion of non-diseased tissues, may still act as useful biomarkers or targets. Another context not considered in this study, but deserving of exploration in a future project, is the impact of epigenetic regulation on retroelement expression and its relevance to different pathological conditions. A technical limitation of this study is our use of Telescope, which does not distinguish between independent and dependent transcription.

## Conclusions

The findings presented herein corroborate previous evidence that retroelements are active and dynamically regulated in normal cells, often exhibiting cell type- or tissue-dependent expression patterns. Based on our reference dataset of normal retroelements, we demonstrated that general retroelement activation during tumorigenesis is a universal phenomenon. Retroelements provide a three-fold larger reservoir of cancer-specific transcripts in comparison to annotated protein-coding genes, highlighting their importance in cancer studies. The activation of specific retroelements during tumorigenesis appears to be influenced by the tissue of origin, possibly due to differences in the epigenetic states of normal cells across tissue systems. Furthermore, analysis of retroelement expression in cancer cells compared to that during embryonic development revealed that two thirds of retroelements reactivated in tumorigenesis are active in early embryonic development, suggesting that tumorigenesis results in the reawakening of pluripotency-associated retroelements whose functions are largely unknown. The results of this study provide a practical resource for future analyses into the functional roles or translational impacts of retroelements in both normal and diseased conditions. To facilitate these studies, we established a catalog containing the retro-transcriptome of 1019 cancer cell lines. Depending on the goal, the retroelements or cell lines can be sorted to identify the optimal retroelement-to-cancer cell line pairing. In addition, we provide a database with all of our raw and processed RNA-sequencing files, *Retroelementdb.com*, enabling researchers to validate and expand upon our findings [21]. This database will be updated on a regular basis, both through our own inquiries and at the request of others.

## Supplementary Information

Supplementary Material 1: Supplemental figures: Figures S1, S2, S3 and S4

Supplementary Material 2: Supplemental tables: Excel file containing additional information too large to fit in a PDF, Supplemental Tables S1-S19

## Data Availability

The retroelement expression data generated in this study can be downloaded from our database, Retroelementdb.com [21]. Additional datasets will be continuously added to expand our current collection.

## References

[1] Sukapan P, Promnarate P, Avihingsanon Y, Mutirangura A, Hirankarn N. Types of *DNA* methylation status of the interspersed repetitive sequences for LINE-1, Alu, HERV-E and HERV-K in the neutrophils from systemic lupus erythematosus patients and healthy controls. J Hum Genet. 2014;59(4):178–88. 24430577 10.1038/jhg.2013.140

[2] Grandi N, Tramontano E. Human endogenous retroviruses are ancient acquired elements still shaping innate immune responses. Front Immunol. 2018;9:2039. 30250470 10.3389/fimmu.2018.02039 PMC6139349

[3] Dupressoir A, Lavialle C, Heidmann T. From ancestral infectious retroviruses to bona fide cellular genes: role of the captured syncytins in placentation. Placenta. 2012;33(9):663–71. 22695103 10.1016/j.placenta.2012.05.005

[4] Kremer D, Schichel T, Forster M, Tzekova N, Bernard C, van der Valk P, et al. Human endogenous retrovirus type W envelope protein inhibits oligodendroglial precursor cell differentiation. Ann Neurol. 2013;74(5):721–32. 23836485 10.1002/ana.23970

[5] Li W, Lee MH, Henderson L, Tyagi R, Bachani M, Steiner J, et al. Human endogenous retrovirus-k contributes to motor neuron disease. Sci Transl Med. 2015;7(307):307ra153. 26424568 10.1126/scitranslmed.aac8201 PMC6344353

[6] Garcia-Montojo M, Doucet-O’Hare T, Henderson L, Nath A. Human endogenous retrovirus-K (HML-2): a comprehensive review. Crit Rev Microbiol. 2018;44(6):715–38. 30318978 10.1080/1040841X.2018.1501345 PMC6342650

[7] Steiner JP, Bachani M, Malik N, DeMarino C, Li W, Sampson K, et al. Human endogenous retrovirus k envelope in spinal fluid of amyotrophic lateral sclerosis is toxic. Ann Neurol. 2022;92(4):545–61. 35801347 10.1002/ana.26452 PMC9489628

[8] Russ E, Iordanskiy S. Endogenous retroviruses as modulators of innate immunity. Pathogens. 2023. 10.3390/pathogens12020162. 36839434 10.3390/pathogens12020162 PMC9963469

[9] Chiappinelli KB, Strissel PL, Desrichard A, Li H, Henke C, Akman B, et al. Inhibiting DNA methylation causes an interferon response in cancer via dsRNA including endogenous retroviruses. Cell. 2015;162(5):974–86. 26317466 10.1016/j.cell.2015.07.011 PMC4556003

[10] Di Giorgio E, Ranzino L, Tolotto V, Dalla E, Burelli M, Gualandi N, et al. Transcription of endogenous retroviruses in senescent cells contributes to the accumulation of double-stranded RNAs that trigger an anti-viral response that reinforces senescence. Cell Death Dis. 2024;15(2):157. 38383514 10.1038/s41419-024-06548-2 PMC10882003

[11] Flockerzi A, Ruggieri A, Frank O, Sauter M, Maldener E, Kopper B, et al. Expression patterns of transcribed human endogenous retrovirus HERV-K(HML-2) loci in human tissues and the need for a HERV transcriptome project. BMC Genomics. 2008;9:354. 18664271 10.1186/1471-2164-9-354 PMC2525661

[12] Schmitt K, Reichrath J, Roesch A, Meese E, Mayer J. Transcriptional profiling of human endogenous retrovirus group HERV-K(HML-2) loci in melanoma. Genome Biol Evol. 2013;5(2):307–28. 23338945 10.1093/gbe/evt010 PMC3590776

[13] Bhardwaj N, Montesion M, Roy F, Coffin JM. Differential expression of HERV-K (HML-2) proviruses in cells and virions of the teratocarcinoma cell line Tera-1. Viruses. 2015;7(3):939–68. 25746218 10.3390/v7030939 PMC4379556

[14] Douville R, Liu J, Rothstein J, Nath A. Identification of active loci of a human endogenous retrovirus in neurons of patients with amyotrophic lateral sclerosis. Ann Neurol. 2011;69(1):141–51. 21280084 10.1002/ana.22149 PMC3052883

[15] Chhangawala S, Rudy G, Mason CE, Rosenfeld JA. The impact of read length on quantification of differentially expressed genes and splice junction detection. Genome Biol. 2015;16(1):131. 26100517 10.1186/s13059-015-0697-y PMC4531809

[16] Tokuyama M, Kong Y, Song E, Jayewickreme T, Kang I, Iwasaki A. ERVmap analysis reveals genome-wide transcription of human endogenous retroviruses. Proc Natl Acad Sci U S A. 2018;115(50):12565–72. 30455304 10.1073/pnas.1814589115 PMC6294949

[17] Bendall ML, de Mulder M, Iniguez LP, Lecanda-Sanchez A, Perez-Losada M, Ostrowski MA, et al. Telescope: Characterization of the retrotranscriptome by accurate estimation of transposable element expression. PLoS Comput Biol. 2019;15(9):e1006453. 31568525 10.1371/journal.pcbi.1006453 PMC6786656

[18] Duarte RRR, Pain O, Bendall ML, de Mulder RM, Marston JL, Selvackadunco S, et al. Integrating human endogenous retroviruses into transcriptome-wide association studies highlights novel risk factors for major psychiatric conditions. Nat Commun. 2024;15(1):3803. 38778015 10.1038/s41467-024-48153-z PMC11111684

[19] Larouche JD, Trofimov A, Hesnard L, Ehx G, Zhao Q, Vincent K, et al. Widespread and tissue-specific expression of endogenous retroelements in human somatic tissues. Genome Med. 2020;12(1):40. 32345368 10.1186/s13073-020-00740-7 PMC7189544

[20] Burn A, Roy F, Freeman M, Coffin JM. Widespread expression of the ancient HERV-K (HML-2) provirus group in normal human tissues. PLoS Biol. 2022;20(10):e3001826. 36256614 10.1371/journal.pbio.3001826 PMC9578601

[21] Retroelementdb. https://Retroelementdb.com. Accessed 17 Feb 2025.

[22] GDC. National Cancer Institute Expression mRNA pipeline. https://docs.gdc.cancer.gov/Data/Bioinformatics_Pipelines/Expression_mRNA_Pipeline/. Accessed on 17 Feb 2025.

[23] Lorenzi L, Chiu HS, Avila Cobos F, Gross S, Volders PJ, Cannoodt R, et al. The RNA atlas expands the catalog of human non-coding RNAs. Nat Biotechnol. 2021;39(11):1453–65. 34140680 10.1038/s41587-021-00936-1

[24] Ardlie KG, Deluca DS, Segre AV, Sullivan TJ, Young TR, Gelfand ET, et al. Human genomics. The Genotype-Tissue Expression (GTEx) pilot analysis: multitissue gene regulation in humans. Science. 2015;348(6235):648–60. 10.1126/science.1262110 PMC4547484 25954001

[25] Gualandi N, Iperi C, Esposito M, Ansaloni F, Gustincich S, Sanges R. Meta-analysis suggests that intron retention can affect quantification of transposable elements from RNA-seq data. Biology. 2022. 10.3390/biology11060826. 35741347 10.3390/biology11060826 PMC9220773

[26] Guo Y, Zhao S, Sheng Q, Guo M, Lehmann B, Pietenpol J, et al. RNAseq by Total RNA Library Identifies Additional RNAs Compared to Poly(A) RNA Library. Biomed Res Int. 2015;2015:862130. 26543871 10.1155/2015/862130 PMC4620295

[27] She J, Du M, Xu Z, Jin Y, Li Y, Zhang D, et al. The landscape of hervRNAs transcribed from human endogenous retroviruses across human body sites. Genome Biol. 2022;23(1):231. 36329469 10.1186/s13059-022-02804-w PMC9632151

[28] Russ E, Mikhalkevich N, Iordanskiy S. Expression of human endogenous retrovirus group K (HERV-K) HML-2 correlates with immune activation of macrophages and type i interferon response. Microbiol Spectr. 2023;11(2):e0443822. 36861980 10.1128/spectrum.04438-22 PMC10100713

[29] Xue B, Sechi LA, Kelvin DJ. Human endogenous retrovirus k (HML-2) in health and disease. Front Microbiol. 2020;11:1690. 32765477 10.3389/fmicb.2020.01690 PMC7380069

[30] Rivas SR, Valdez MJM, Govindarajan V, Seetharam D, Doucet-O’Hare TT, Heiss JD, et al. The role of HERV-K in cancer stemness. Viruses. 2022. 10.3390/v14092019. 36146825 10.3390/v14092019 PMC9504571

[31] Ryder PV, Lerit DA. RNA localization regulates diverse and dynamic cellular processes. Traffic. 2018;19(7):496–502. 29653028 10.1111/tra.12571 PMC6003861

[32] Ferguson CM, Echeverria D, Hassler M, Ly S, Khvorova A. Cell type impacts accessibility of mRNA to silencing by RNA interference. Mol Ther Nucleic Acids. 2020;21:384–93. 32650236 10.1016/j.omtn.2020.06.006 PMC7340969

[33] Kawamura Y, Sanchez Calle A, Yamamoto Y, Sato TA, Ochiya T. Extracellular vesicles mediate the horizontal transfer of an active LINE-1 retrotransposon. J Extracell Vesicles. 2019;8(1):1643214. 31448067 10.1080/20013078.2019.1643214 PMC6691892

[34] DeMarino C, Nath A, Zhuang Z, Doucet-O’Hare TT. Does the interplay between human endogenous retrovirus K and extracellular vesicles contribute to aging? Extracell Vesicles Circ Nucleic Acids. 2023;4(4):548–56. 10.20517/evcna.2023.45 PMC11007738 38606283

[35] Fuke H, Ohno M. Role of poly (A) tail as an identity element for mRNA nuclear export. Nucleic Acids Res. 2008;36(3):1037–49. 18096623 10.1093/nar/gkm1120 PMC2241894

[36] Heslin DJ, Murcia P, Arnaud F, Van Doorslaer K, Palmarini M, Lenz J. A single amino acid substitution in a segment of the CA protein within Gag that has similarity to human immunodeficiency virus type 1 blocks infectivity of a human endogenous retrovirus K provirus in the human genome. J Virol. 2009;83(2):1105–14. 19004950 10.1128/JVI.01439-08 PMC2612375

[37] Ruprecht K, Ferreira H, Flockerzi A, Wahl S, Sauter M, Mayer J, et al. Human endogenous retrovirus family HERV-K(HML-2) RNA transcripts are selectively packaged into retroviral particles produced by the human germ cell tumor line Tera-1 and originate mainly from a provirus on chromosome 22q11.21. J Virol. 2008;82(20):10008–16. 18684837 10.1128/JVI.01016-08 PMC2566279

[38] Suntsova M, Gogvadze EV, Salozhin S, Gaifullin N, Eroshkin F, Dmitriev SE, et al. Human-specific endogenous retroviral insert serves as an enhancer for the schizophrenia-linked gene PRODH. Proc Natl Acad Sci U S A. 2013;110(48):19472–7. 24218577 10.1073/pnas.1318172110 PMC3845128

[39] Liu K, Lin B, Zhao M, Yang X, Chen M, Gao A, et al. The multiple roles for Sox2 in stem cell maintenance and tumorigenesis. Cell Signal. 2013;25(5):1264–71. 23416461 10.1016/j.cellsig.2013.02.013 PMC3871517

[40] Guo JC, Fang SS, Wu Y, Zhang JH, Chen Y, Liu J, et al. CNIT: a fast and accurate web tool for identifying protein-coding and long non-coding transcripts based on intrinsic sequence composition. Nucleic Acids Res. 2019;47(W1):W516–22. 31147700 10.1093/nar/gkz400 PMC6602462

[41] Uhlen M, Fagerberg L, Hallstrom BM, Lindskog C, Oksvold P, Mardinoglu A, et al. Proteomics. Tissue-based map of the human proteome. Science. 2015;347(6220):1260419. 25613900 10.1126/science.1260419

[42] Kim WT, Ryu CJ. Cancer stem cell surface markers on normal stem cells. BMB Rep. 2017;50(6):285–98. 28270302 10.5483/BMBRep.2017.50.6.039 PMC5498139

[43] Gaggianesi M, Di Franco S, Pantina VD, Porcelli G, D’Accardo C, Verona F, et al. Messing up the cancer stem cell chemoresistance mechanisms supported by tumor microenvironment. Front Oncol. 2021;11:702642. 34354950 10.3389/fonc.2021.702642 PMC8330815

[44] Li Y, Rogoff HA, Keates S, Gao Y, Murikipudi S, Mikule K, et al. Suppression of cancer relapse and metastasis by inhibiting cancer stemness. Proc Natl Acad Sci U S A. 2015;112(6):1839–44. 25605917 10.1073/pnas.1424171112 PMC4330785

[45] Carter TA, Singh M, Dumbovic G, Chobirko JD, Rinn JL, Feschotte C. Mosaic cis-regulatory evolution drives transcriptional partitioning of HERVH endogenous retrovirus in the human embryo. eLife. 2022;11:e76257. 10.7554/eLife.76257 PMC8912925 35179489

[46] Santoni FA, Guerra J, Luban J. Herv-H RNA is abundant in human embryonic stem cells and a precise marker for pluripotency. Retrovirology. 2012;9:111. 23253934 10.1186/1742-4690-9-111 PMC3558390

[47] Zhang Y, Li T, Preissl S, Amaral ML, Grinstein JD, Farah EN, et al. Transcriptionally active HERV-H retrotransposons demarcate topologically associating domains in human pluripotent stem cells. Nat Genet. 2019;51(9):1380–8. 31427791 10.1038/s41588-019-0479-7 PMC6722002

[48] Ohnuki M, Tanabe K, Sutou K, Teramoto I, Sawamura Y, Narita M, et al. Dynamic regulation of human endogenous retroviruses mediates factor-induced reprogramming and differentiation potential. Proc Natl Acad Sci U S A. 2014;111(34):12426–31. 25097266 10.1073/pnas.1413299111 PMC4151758

[49] Lu X, Sachs F, Ramsay L, Jacques PE, Goke J, Bourque G, et al. The retrovirus HERVH is a long noncoding RNA required for human embryonic stem cell identity. Nat Struct Mol Biol. 2014;21(4):423–5. 24681886 10.1038/nsmb.2799

[50] Cancer Genome Atlas Research N, Weinstein JN, Collisson EA, Mills GB, Shaw KR, Ozenberger BA, et al. The cancer genome atlas pan-cancer analysis project. Nat Genet. 2013;45(10):1113–20. 24071849 10.1038/ng.2764 PMC3919969

[51] Kitsou K, Lagiou P, Magiorkinis G. Human endogenous retroviruses in cancer: oncogenesis mechanisms and clinical implications. J Med Virol. 2023;95(1):e28350. 36428242 10.1002/jmv.28350 PMC10108094

[52] Wang J, Han GZ. Frequent retroviral gene co-option during the evolution of vertebrates. Mol Biol Evol. 2020;37(11):3232–42. 32667990 10.1093/molbev/msaa180

[53] Esnault C, Priet S, Ribet D, Vernochet C, Bruls T, Lavialle C, et al. A placenta-specific receptor for the fusogenic, endogenous retrovirus-derived, human syncytin-2. Proc Natl Acad Sci U S A. 2008;105(45):17532–7. 18988732 10.1073/pnas.0807413105 PMC2582322

[54] Blanco-Melo D, Gifford RJ, Bieniasz PD. Co-option of an endogenous retrovirus envelope for host defense in hominid ancestors. eLife. 2017;6:e22519. 10.7554/eLife.22519 PMC5388530 28397686

[55] Statello L, Guo CJ, Chen LL, Huarte M. Gene regulation by long non-coding RNAs and its biological functions. Nat Rev Mol Cell Biol. 2021;22(2):96–118. 33353982 10.1038/s41580-020-00315-9 PMC7754182

[56] Logotheti S, Stiewe T, Georgakilas AG. The role of human endogenous retroviruses in cancer immunotherapy of the post-COVID-19 world. Cancers (Basel). 2023. 10.3390/cancers15225321. 38001581 10.3390/cancers15225321 PMC10669663

[57] Ng KW, Boumelha J, Enfield KSS, Almagro J, Cha H, Pich O, et al. Antibodies against endogenous retroviruses promote lung cancer immunotherapy. Nature. 2023;616(7957):563–73. 37046094 10.1038/s41586-023-05771-9 PMC10115647

[58] Adhit KK, Wanjari A, Menon S, K S. Liquid Biopsy: An Evolving Paradigm for Non-invasive Disease Diagnosis and Monitoring in Medicine. Cureus. 2023;15(12):e50176. 10.7759/cureus.50176 PMC10772356 38192931

[59] Mastora E, Christodoulaki A, Papageorgiou K, Zikopoulos A, Georgiou I. Expression of retroelements in mammalian gametes and embryos. In Vivo. 2021;35(4):1921–7. 34182464 10.21873/invivo.12458 PMC8286539

[60] Kohlrausch FB, Berteli TS, Wang F, Navarro PA, Keefe DL. Control of LINE-1 expression maintains genome integrity in germline and early embryo development. Reprod Sci. 2022;29(2):328–40. 33481218 10.1007/s43032-021-00461-1

[61] Shah AH, Rivas SR, Doucet-O'Hare TT, Govindarajan V, DeMarino C, Wang T, et al. Human endogenous retrovirus K contributes to a stem cell niche in glioblastoma. J Clin Invest. 2023;133(13):e167929. 10.1172/JCI167929 PMC10313366 37395282

[62] Reyes-Gopar H, Marston JL, Singh B, Greenig M, Lin J, Ostrowski MA, et al. A single-cell transposable element atlas of human cell identity. bioRxiv. 2023. 10.1101/2023.12.28.573568. 40543500 10.1016/j.crmeth.2025.101086 PMC12296447

