# Supplementary material for: Strict retroelement regulation is frequently lost following cancer transformation and generates a promising reservoir of cancer biomarkers
Source: Mob DNA. 2025 Oct 3;16:37. doi: 10.1186/s13100-025-00376-7 (PMC12495673; doi:10.1186/s13100-025-00376-7)
Supplement: Supplementary file 1 — Supplementary Material 1: Supplemental figures: Figures S1, S2, S3 and S4 [file 13100_2025_376_MOESM1_ESM.pptx]

## Slide 1
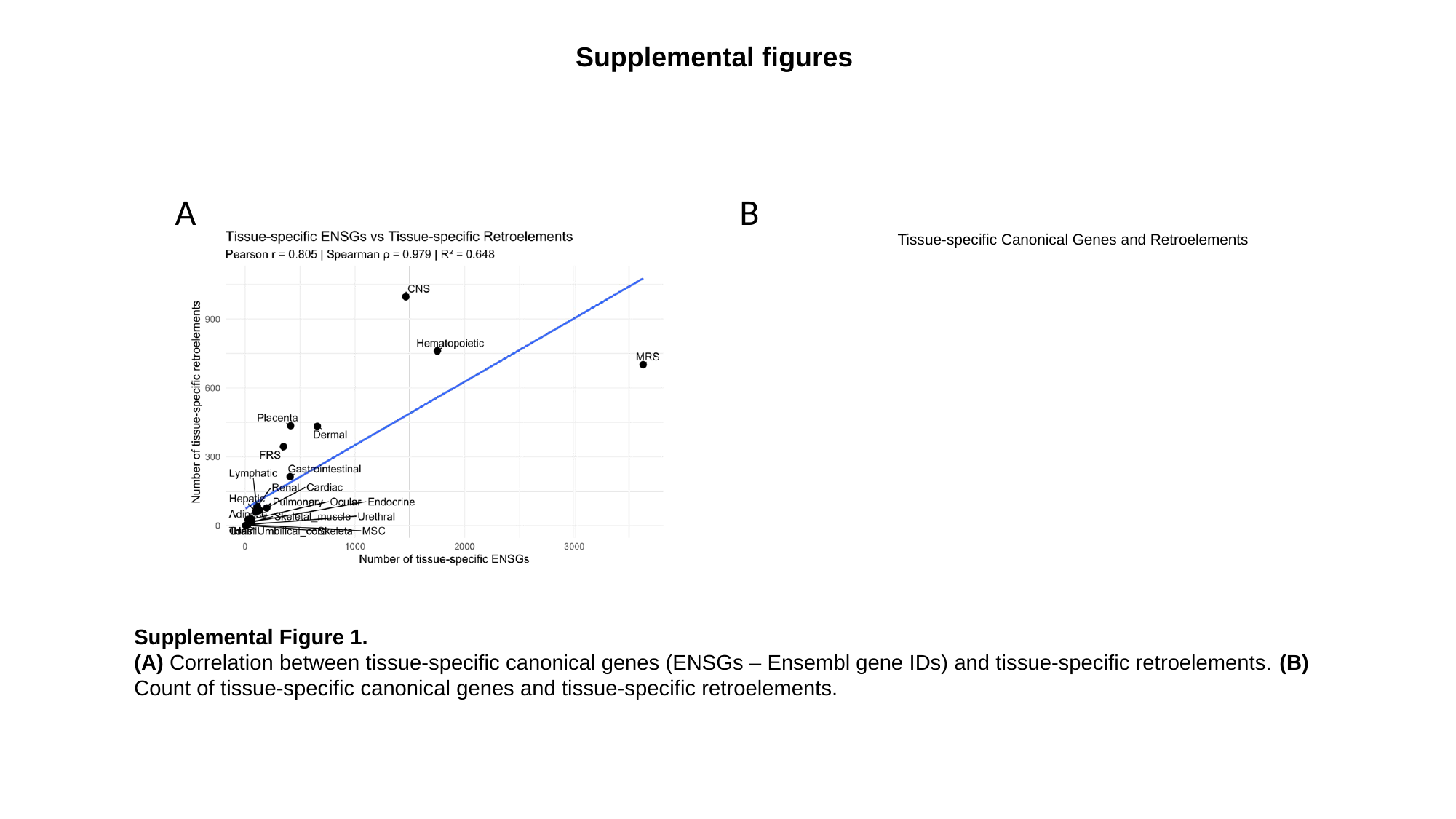

Supplemental figures
A
B
Tissue-specific Canonical Genes and Retroelements
Supplemental Figure 1.
(A) Correlation between tissue-specific canonical genes (ENSGs – Ensembl gene IDs) and tissue-specific retroelements. (B) Count of tissue-specific canonical genes and tissue-specific retroelements.

## Slide 2
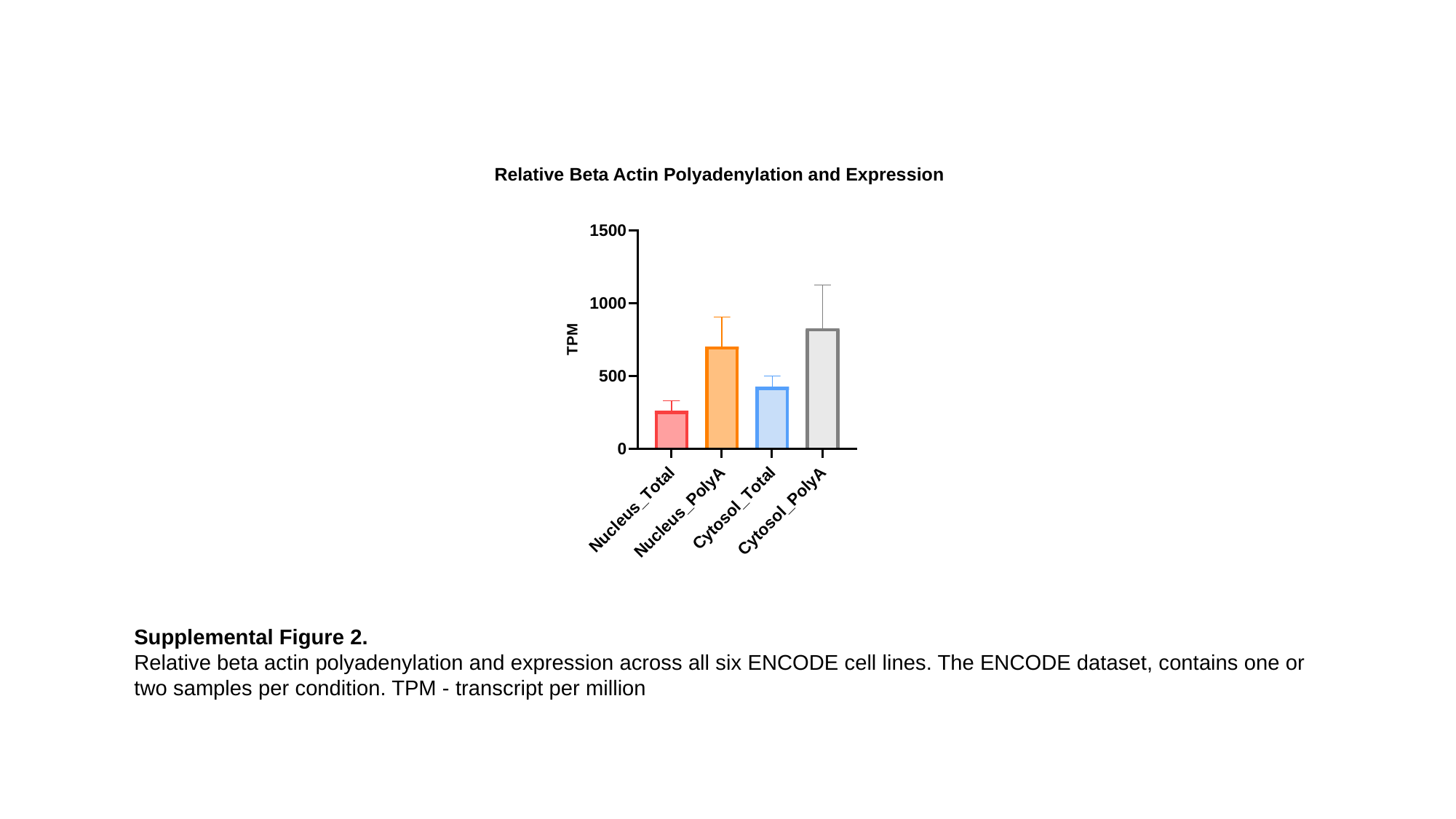

Relative Beta Actin Polyadenylation and Expression
Supplemental Figure 2.
Relative beta actin polyadenylation and expression across all six ENCODE cell lines. The ENCODE dataset, contains one or two samples per condition. TPM - transcript per million

## Slide 3
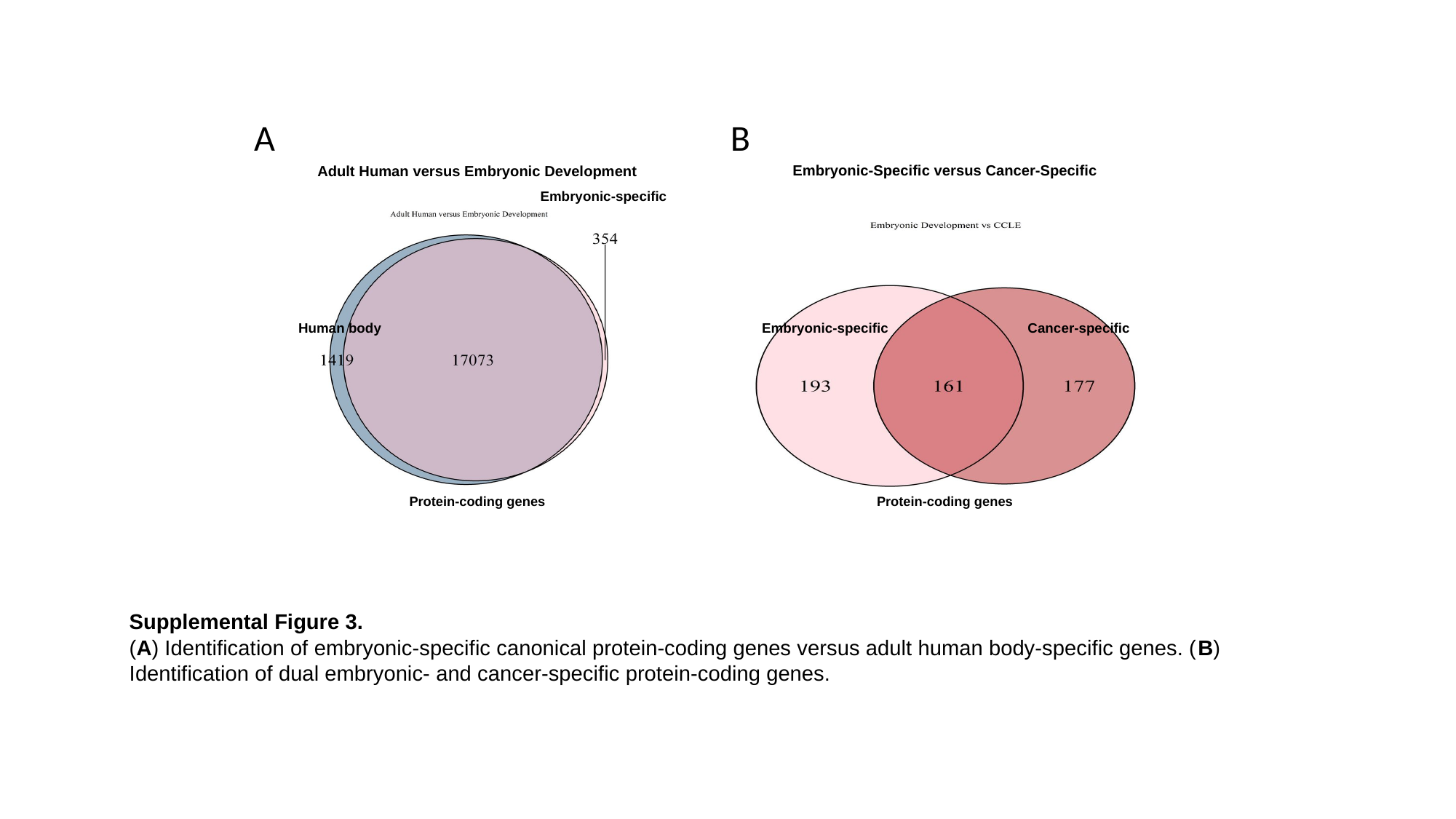

A
B
Embryonic-Specific versus Cancer-Specific
Adult Human versus Embryonic Development
Embryonic-specific
Embryonic-specific
Cancer-specific
Human body
Protein-coding genes
Protein-coding genes
Supplemental Figure 3.
(A) Identification of embryonic-specific canonical protein-coding genes versus adult human body-specific genes. (B) Identification of dual embryonic- and cancer-specific protein-coding genes.

## Slide 4
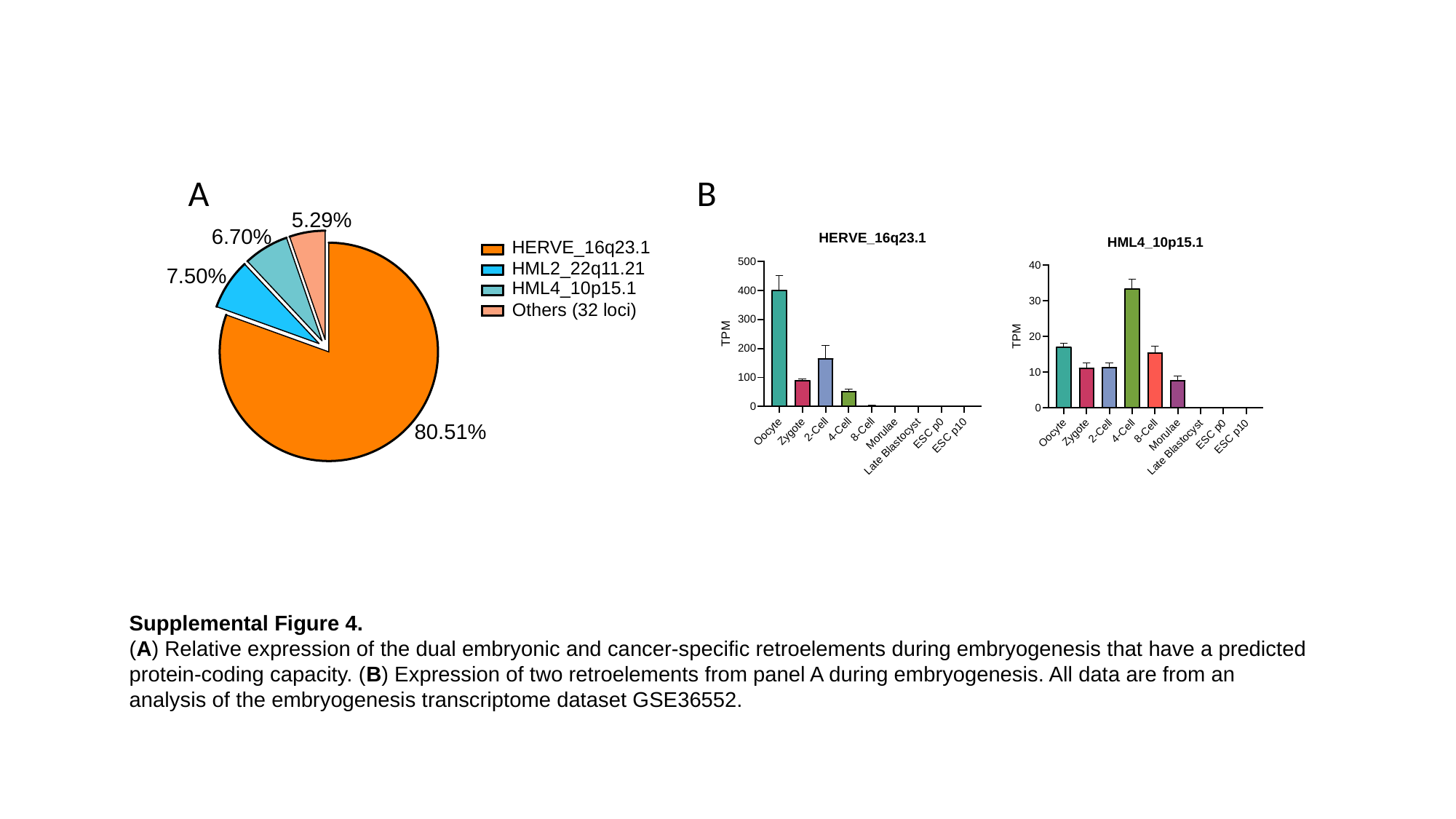

A
B
5.29%
6.70%
HERVE_16q23.1
HML2_22q11.21
7.50%
HML4_10p15.1
Others (32 loci)
80.51%
Supplemental Figure 4.
(A) Relative expression of the dual embryonic and cancer-specific retroelements during embryogenesis that have a predicted protein-coding capacity. (B) Expression of two retroelements from panel A during embryogenesis. All data are from an analysis of the embryogenesis transcriptome dataset GSE36552.
